# Worldwide Phylogenetic Distributions and Population Dynamics of the Genus *Histoplasma*

**DOI:** 10.1371/journal.pntd.0004732

**Published:** 2016-06-01

**Authors:** Marcus de M. Teixeira, José S. L. Patané, Maria L. Taylor, Beatriz L. Gómez, Raquel C. Theodoro, Sybren de Hoog, David M. Engelthaler, Rosely M. Zancopé-Oliveira, Maria S. S. Felipe, Bridget M. Barker

**Affiliations:** 1 Division of Pathogen Genomics, Translational Genomics Research Institute-North, Flagstaff, Arizona, United States of America; 2 Department of Cell Biology, University of Brasília, Brasilia, Brazil; 3 Department of Biochemistry, University of São Paulo, São Paulo, Brazil; 4 Department of Microbiology and Parasitology, National Autonomous University of Mexico, Mexico City, Mexico; 5 Corporación para Investigaciones Biológicas (CIB), Medellín, Colombia; 6 Department of Cell Biology and Genetics/ Institute of Tropical Medicine, Federal University of Rio Grande do Norte, Natal, Brazil; 7 CBS-KNAW Fungal Biodiversity Centre, Utrecht, Netherlands; 8 Mycology Laboratory, National Institute of Infectology Evandro Chagas, Oswaldo Cruz Foundation, Rio de Janeiro, Brazil; University of California San Diego School of Medicine, UNITED STATES

## Abstract

**Background:**

*Histoplasma capsulatum* comprises a worldwide complex of saprobiotic fungi mainly found in nitrogen/phosphate (often bird guano) enriched soils. The microconidia of *Histoplasma* species may be inhaled by mammalian hosts, and is followed by a rapid conversion to yeast that can persist in host tissues causing histoplasmosis, a deep pulmonary/systemic mycosis. *Histoplasma capsulatum sensu lato* is a complex of at least eight clades geographically distributed as follows: Australia, Netherlands, Eurasia, North American classes 1 and 2 (NAm 1 and NAm 2), Latin American groups A and B (LAm A and LAm B) and Africa. With the exception of the Eurasian cluster, those clades are considered phylogenetic species.

**Methodology/Principal Findings:**

Increased *Histoplasma* sampling (n = 234) resulted in the revision of the phylogenetic distribution and population structure using 1,563 aligned nucleotides from four protein-coding regions. The LAm B clade appears to be divided into at least two highly supported clades, which are geographically restricted to either Colombia/Argentina or Brazil respectively. Moreover, a complex population genetic structure was identified within LAm A clade supporting multiple monophylogenetic species, which could be driven by rapid host or environmental adaptation (~0.5 MYA). We found two divergent clades, which include Latin American isolates (newly named as LAm A1 and LAm A2), harboring a cryptic cluster in association with bats.

**Conclusions/Significance:**

At least six new phylogenetic species are proposed in the *Histoplasma* species complex supported by different phylogenetic and population genetics methods, comprising LAm A1, LAm A2, LAm B1, LAm B2, RJ and BAC-1 phylogenetic species. The genetic isolation of *Histoplasma* could be a result of differential dispersion potential of naturally infected bats and other mammals. In addition, the present study guides isolate selection for future population genomics and genome wide association studies in this important pathogen complex.

## Author Summary

Histoplasmosis is a potentially severe fungal disease of mammals caused by *Histoplasma capsulatum*. The highest incidence of the disease is reported on the American continent, and approximately 30% of HIV and histoplasmosis co-infections are fatal. Previous studies have suggested at least 7 phylogenetic species within *H*. *capsulatum*, however by increasing taxon sampling and using different phylogenetic and population genetic methods, we detect at least 5 additional phylogenetic species within Latin America (LAm A1, LAm A2, LAm B1, LAm B2, RJ and BAC-1). These phylogenetic species are nested in the former LAm A clade. We found evidence that bats may be a cause of speciation in *Histoplasma*, as well-supported monophyletic clades were found in association with different species of bats. The radiation of the Latin American *H*. *capsulatum* species took a place around 5 million years ago, which is consistent with the radiation and diversification of bat species. Previous phylogenetic distribution of *Histoplasma* is upheld and strong support is indicated for the species delineation and evolution of this important pathogen.

## Introduction

*Histoplasma capsulatum sensu lato* are dimorphic fungal species found in a filamentous form in the environment as saprobiotic-geophilic microorganisms [1]. These species encompass a globally distributed complex of fungi, which are mainly found in nitrogen/phosphate-enriched soils associated with bird and bat guano [2, 3]. In addition, moderate temperatures (18–28°C), constant humidity (>60%), and a low light environment characterize suitable ecological conditions for fungal growth [4, 5]. *Histoplasma* species in the saprobic mycelial phase at less than 35°C can produce asexual microconidia and macroconidia [6]. Microconidia, macroconidia or fragmented hyphal cells may be inhaled by various vertebrate species, including humans, and upon reaching the alveoli undergo a rapid conversion to yeast cells that can persist in host lungs and may disseminate to other tissues causing histoplasmosis [5]. The pathogenic yeasts are ovoid thick-walled cells that can be cultured in cysteine-enriched blood or brain-heart infusion media (BHI) at 37°C or in infected tissues [5]. In addition, *Histoplasma* utilize a heterothallic mating system where opposite mating type strains can complete the sexual cycle. Ascocarps are produced by hyphal constriction and coiling of opposite mating type cells thus forming the gymnothecia harboring meiospores [7].

Histoplasmosis was first reported by Samuel Darling in 1905–1906 in a patient from Martinique [8]. The spectrum of histoplasmosis varies from asymptomatic infection or mild illness to deep pulmonary and/or systemic mycosis. Severe clinical manifestations occur in less than 1% of patients [9–11]. Immunocompetent humans may acquire the disease tilling soil, visiting caves, building, cleaning old houses or bird roosting sites or even cutting down trees [12]. Among immuncompromised population with acquired cellular immunity impairment (HIV), the disease is responsible for high rates of morbidity and mortality [13]. In addition, with the increase of immunosuppressive therapy due to transplants and other chronic inflammatory disorders, disseminated histoplasmosis is becoming more frequent and is geographically expanding [14–16]. Autochthonous outbreaks of histoplasmosis have been reported in the latitudes 54° North and 38° South [17]. In patients with an impaired immune system, the disease is mostly fatal without an early diagnosis and proper treatment. For example, in Latin America about 30% of HIV infected people may die from histoplasmosis [4, 18].

Outbreaks are often reported with *Histoplasma*-contaminated soils, commonly in the presence of bat or bird guano. Birds are infected with *H*. *capsulatum* sporadically, however they could play role in its dispersal [19]. In endemic areas of histoplasmosis throughout the Americas, bats are often infected with this fungus [20, 21]. In addition to bats, the fungus has been detected in wild mammals such as non-human primates (e.g., baboons) [22, 23], mustelids (e.g., badgers and northern sea otter) [24–26], procyonids (e.g., raccoons) [27], as well as in domesticated animals such as equines [28, 29], felines [30, 31] and canines [32, 33]. This broad host range and tight association with vertebrates suggests that mammals play an important role in the speciation and dispersal mechanisms of this species complex.

*Histoplasma capsulatum* is a cosmopolitan fungus, and epidemiological knowledge has been improved by serology, culturing and molecular-based diagnostic methods [13, 34]. The endemicity of histoplasmosis varies from low (Europe and Oceania), to moderate (Africa and South Asia), to high (Americas) prevalence areas. The Midwestern and Southeastern regions of the United States, specifically the Ohio, St Lawrence and Mississippi river regions are considered highly endemic [11]. In Latin America, the high prevalence areas range from Uruguay to Mexico, mostly in countries with a moderate climate and constant humidity [4]. In Africa, two well-delimited areas are considered endemic—southern Africa, which includes South Africa, Tanzania and Zimbabwe, and western/central Africa [35]. In southern Asia, histoplasmosis is found in China, India and Thailand based on very few clinical reports; however, skin test surveys suggest that the fungus also occurs in Malaysia, Indonesia, Myanmar and the Philippines [36, 37]. Epidemiological surveys suggest that under-surveyed areas of the disease can have moderate to high levels of *Histoplasma* natural infections and the impact of the disease outside of the current known endemic regions should be investigated.

Based on phenotypic characteristics (host, morphology and pathogenicity), the genus *Histoplasma* was split into three varieties: *H*. *capsulatum var*. *capsulatum*, *H*. *capsulatum var*. *duboisii* and *H*. *capsulatum var*. *farciminosum*. The *capsulatum* var. is the most broadly dispersed, the lungs are the main compromised organs, and in isolated cases the phagocytic mononuclear system is involved in disseminated forms. The var. *H*. *duboisii* is restricted to tropical areas in Africa, causing cutaneous, subcutaneous and bone lesions and the var. *farciminosum* isolates are known to occur in Europe, Northern Africa, India and Southern Asia, commonly infecting horses and mules [5]. Advances in molecular genotyping methods have led to a closer investigation of *H*. *capsulatum sensu stricto*. Techniques such as Restriction Fragment Length Polymorphisms (RFLP), DNA hybridization, Random Amplified Polymorphic DNA (RAPD) and ITS1/2 sequencing all revealed high genetic diversity in *Histoplasma*, with some indication of geographical association [21]. Population structure based on molecular genotyping suggested different patterns of genetic variation among *H*. *capsulatum* populations, and that both recombination and clonal reproduction might occur [38]. Applying the genealogic concordance for phylogenetic species recognition (GCPSR) method [39, 40] for *H*. *capsulatum sensu stricto* using four partial coding loci in 137 isolates distributed across 25 countries, Kasuga and colleagues suggested at least 8 clades with 7 phylogenetic species: North America clade 1 (NAm 1), North America clade 2 (NAm 2), Latin America clade A (LAm A), Latin America clade B (LAm B), Australia, Netherlands, Eurasia, and Africa, where the Eurasian clade was not considered as a phylogenetic species by these authors [40]. This study improved our understanding of cryptic speciation and epidemiology for *Histoplasma*, however because of the ubiquitous worldwide distribution and broad host range, a complete understanding of the phylogenetic relationships of *H*. *capsulatum sensu stricto* is lacking. In addition, regional genetic diversity of this pathogen has been assessed, but a meta-analysis of these data has not been completed [41–44].

Therefore, we applied GCPSR and population genetic meta-analysis using four genetic markers across 234 globally-distributed taxa to better understand the phylogenetic structure of *H*. *capsulatum sensu stricto* [39]. Our main questions were: (i) What is the phylogenetic species distribution for *H*. *capsulatum sensu stricto*? (ii) Do GCPSR and population structure methods reveal similar patterns of species delineation in *Histoplasma*? (iii) Is there any evidence for host-derived speciation in *H*. *capsulatum*? (iv) Does recombination occur between and within populations, and is there evidence for introgression or gene flow between populations?

## Methods

### Phylogenetic and haplotype network analyses

Sequences of partial protein-coding loci used in this study were ADP-ribosylation factor (*arf*), H antigen precursor (*H-anti*), delta-9 fatty acid desaturase (*ole1*) and alpha-tubulin (*tub1*) from 234 isolates that were retrieved from GENBANK and TreeBASE and are listed in **S1 Table**. To increase sample size and phylogenetic signal we collected all *Histoplasma* isolate genome data that had at least one deposited sequence of the aforementioned studied loci. Recombining sites were removed from each individual gene alignment using RDP4 software, which could cause systematic errors in phylogenetic tree estimation [45]. For the final concatenated data set containing the four loci, missing sequences were treated as missing data for the final matrix totaling 234 taxa. Sequences were aligned using Muscle algorithm within MEGA 6.0 with default options [46]. Nucleotide substitution of best-fit model for each locus were statistically selected using jModelTest 2.0 [47]. Phylogenetic analyses were conducted by Bayesian Inference (BI) and Maximum Likelihood (ML) methods.

ML trees were inferred by fast and effective stochastic algorithm implemented in IQ-TREE v1.3.5 software [48]. Branch support was inferred by 1,000 non-parametric bootstrap pseudoreplicates [49]. BI was used to infer the phylogeny altogether as well divergence dating using BEAST v1.8.2 [50] based on conservative intervals of nucleotide substitutions rates and dates (0.00043–0.00656 subst/site/lineage/My; 0.0–15 mya) that encompass values obtained by Kasuga *et al*. [40] (0.0086–0.00432 s/s/l/My; 3.2–13.0 Ma). BI branch support was inferred by posterior probabilities [51]. The presence of saturation in the sequences was tested using DAMBE 5 [52] to infer if a strict molecular clock would be reasonable or whether a relaxed clock should be implemented. As this test did not detect saturation, we proceeded with the use of a strict molecular clock, using rate and date priors (parameters clock.rate and treeModel.rootHeight) for the ranges above. The runs were implemented under a coalescent framework, assuming the Extended Bayesian Skyline Plot [53] population model in which the effective population size is allowed to change throughout the tree time frame. Each run was performed twice for 100M generations (or longer in case of lack of convergence). Convergence of all runs (including the ones described below), parameter effective sample sizes (ESSs), burn-in determination, and 95% highest posterior densities (HPDs) of parameters were done in Tracer v1.6 [54]. Consolidation of run results was done in LogCombiner v1.8.2 [50], after removing the burn-in.

Haplotype networks were built using 202 strains through harboring sequences for each one of the four loci analyzed to visualize differences and diversity among *Histoplasma* worldwide populations. Thirty isolates were excluded from haplotype analysis due missing sequence information for at least one locus to avoid bias [55]. The haplotype distribution and diversity of the four analyzed loci were estimated using DnaSP v5 and used for input in Network 4.6.1.6 (fluxus-engineering.com). Gaps and missing data were excluded from the analysis. Median-joining networks [56] for each locus were obtained and visualized in Network 4.6.1.6.

### Population structure in *H*. *capsulatum*

Genetic differentiation and neutrality tests between populations of *H*. *capsulatum* were calculated using Arlequin v. 3.11 [57]. Input haplotype files containing 202 taxa were generated using DnaSP v5, and Φ_ST_ was calculated. The statistical significance of phi-statistic was tested based on 1000 permutations using default settings. The effects of natural selection on molecular polymorphisms among the *Histoplasma* sequences was assessed using Tajima’s *D* [58] and Fu’s *F*_*S*_ neutrality tests [59]. Population structure within *Histoplasma* was also assessed by SNP matrix calculated from the four analyzed loci with Bayesian Analysis of Population Structure (BAPS) 6.0 [60]. BAPS uses a Bayesian approach under an admixture model to infer the true number of natural populations in a single run [61, 62]. It differs from STRUCTURE [63] in methodology to address linked markers (as is the case for SNPs closely linked to one another in the same gene). It proceeds in two phases: first, it estimates K (inferred number of natural populations) under a no-admixture model; then, using these *a priori* populations, it estimates the probability (0.0–1.0) of each individual being from each of these K populations. We assumed a model in which individual SNPs from the same gene are considered linked but the four genes are assumed to be unlinked. Ten runs were performed for each of the two phases (no-admixture and admixture) and results automatically assembled into a single bar graph, where each bar represents an individual and colors represent the probability of it being assigned to a population. An output file is also generated reporting these individual probabilities for each individual.

Inference of the population tree was done in *Beast v1.8.2 assuming a species tree model, therefore assuming that each gene tree evolved within the actual species tree, but possibly having different topologies due to incomplete lineage sorting. Only individuals with P > = 0.95 of pertaining to a single population as inferred by BAPS in the previous step were included (for a total of 193 individuals included), therefore minimizing artifact branches in the species tree due to shared ancestry. The model for each of the genes was the same obtained by jModelTest previously to the ML search (see above). A birth-death model with incomplete sampling was chosen as the prior for the species tree topology, with rates (clock.rate) fixed at a relative rate of one. Two MCMC chains were used. Number of generations, assessment of convergence, and generation of the clade credibility tree followed the same steps previously described.

## Results/Discussion

### Worldwide distribution of *Histoplasma*

**Fig 1:**
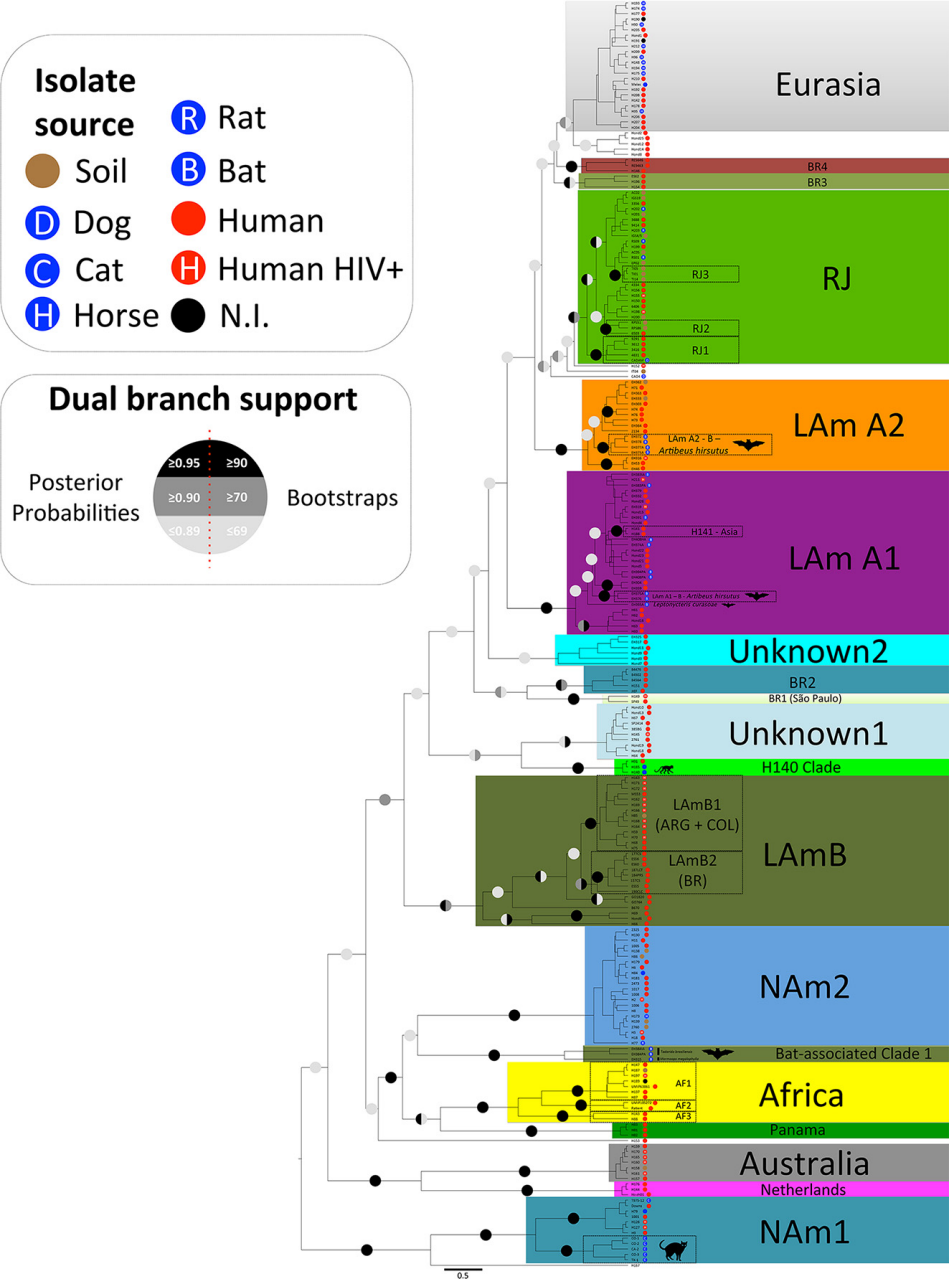
Maximum Likelihood (ML) tree of *Histoplasma capsulatum* generated by IQ-TREE software for 232 taxa through 4 different loci (*arf*, *ole1*, *tub* and *anti-H* loci) reveals at least monophyletic braches as following: NAm 1, NAm 2, RJ, LAm B, NAm LAm A1, LAm A2, BR1-4, and Cluster 6 containing Netherlands, Panama, Africa, Australia and BAC1. Dual branch support, inferred by non-parametric bootstrap for ML analysis, combined with posterior probabilities obtained for the BI analysis, was added to the branches. Monophyletic branches that were supported by two methods (Bootstrap≥70/Posterior Probabilities≥0.95) were designated high confidence clades. We also identified possible in-group variation that may be associated with specific niches. Low supported clades such as Eurasia, Unknown 1 and Unknown 2 were detected but do not follow our monophyletic branches supporting criteria.

Our phylogenetic analysis of *H*. *capsulatum sensu stricto* revealed at least 17 cryptic phylogenetic species supported by bootstrap and posterior probabilities (**Fig 1**). The majority of novel phylogenetic species identified are nested within LAm A (LAm A1, LAm A2 and RJ) and LAm B (LAm B1 and LAm B2) species as proposed previously [40]. However the basal clades diagnosed by phylogenetic analyses display low bootstrap and posterior probability values, and the four analyzed loci did not resolve those branches (**Fig 1**). Low supported clades such as Unknown 1 and Unknown 2 harboring Latin American isolates as well the previously described Eurasia clade were also identified. Low-resolution branches could be explained by either rapid diversification of *Histoplasma*, or accelerated mutation rates in Central-South America. Alternatively, sampling of four loci and the numbers of representatives of each population were insufficient to resolve phylogenies.

Admixture analysis inferred by Bayesian structuring methods reveals at least six populations as follows: Cluster 1 representing the phylogenetic species NAm 1, Cluster 2 representing the phylogenetic species RJ, Cluster 3 containing LAm B, Cluster 4 constituted by phylogenetic species NAm 2, Cluster 5 constituted by LAm A1, LAm A2, BR1-4, and the paraphyletic low supported clades Eurasia, Unknown 1 and Unknown 2, and Cluster 6 containing Netherlands, Panama, Africa, Australia and BAC1. To further explore the cryptic species of *H*. *capsulatum sensu stricto*, monophyletic branches and population structures were investigated individually to reconstruct the biogeographic history of this important pathogen.

### North American *Histoplasma capsulatum* populations

*Histoplasma capsulatum* is endemic to North America, and mainly found in the central and eastern regions of the United States through Mississippi, St Lawrence and Ohio River valleys. Beyond humans, histoplasmosis is the second most common systemic fungal disease of cats in the USA [33]. About 60% to 90% of people who live in endemic areas in the US positively react to histoplasmin skin tests (HST) [64]. Among the elderly, the disease incidence varies from 3.4 to 6.1 cases per 100,000 individuals with highest rates in the Midwestern USA. Serological data show that the fungus naturally infects people in Canada, however rates are relatively low. The sensitivity reaction to HST ranges from 0 to 68% among tested Canadian populations, with higher prevalence of autochthonous infections in Ontario and Quebec provinces [65]. Recent expansion of autochthonous infections were detected in Montana [66] and an outbreak was reported in the Quebec province of Canada among employees from a demolition company [67]. Several outbreaks have been demonstrated in North America [68] and studies comparing genotypes of infectious strains are encouraged, as this could assist with predicting potential for infection. Despite the high incidence of this mycosis in North America little is known about the genetic background of the causative agents of histoplasmosis, relationships with different hosts, and variation in clinical presentation of the disease.

**Fig 2:**
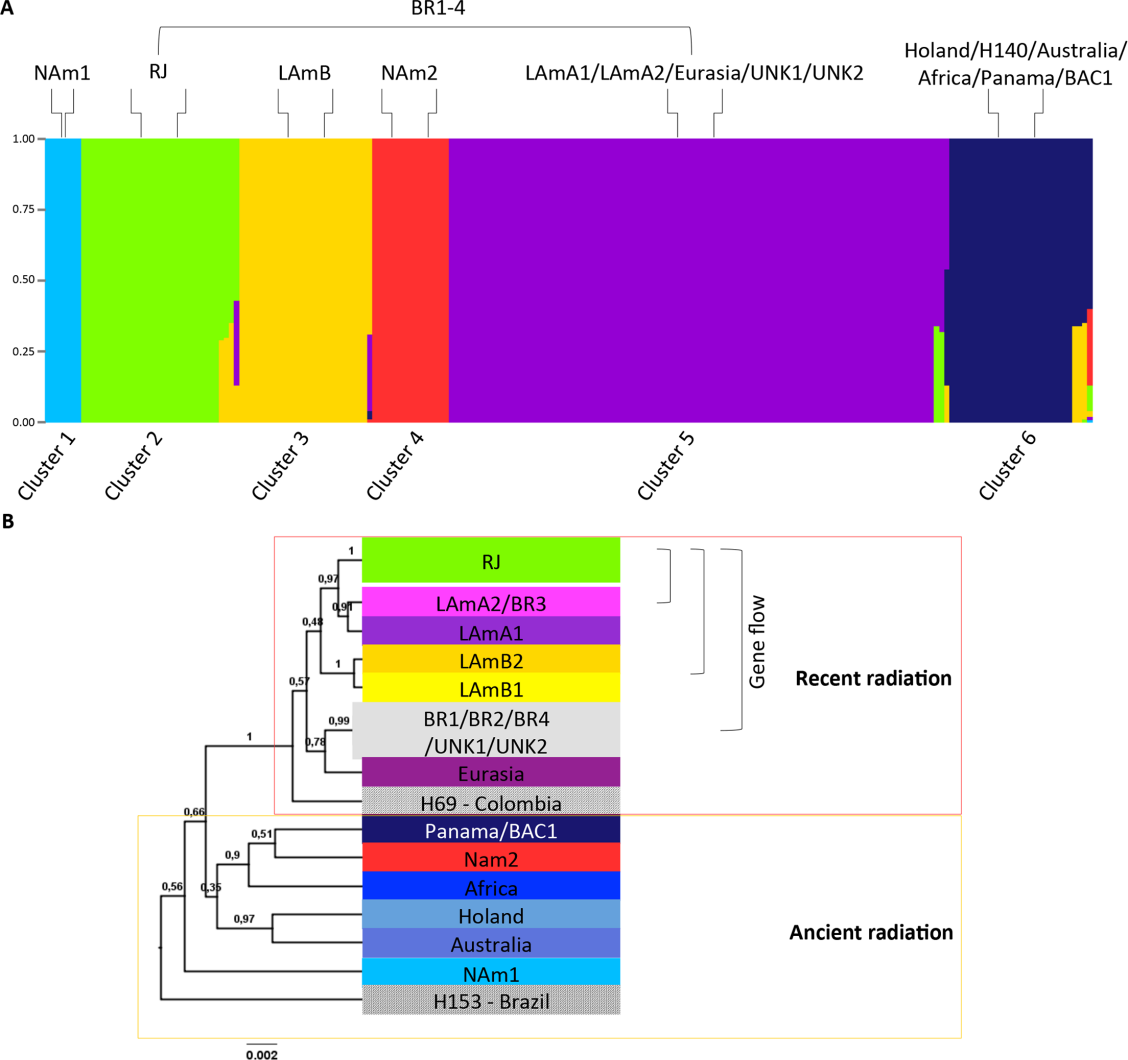
Population structure of *Histoplasma capsulatum* deduced by Bayesian Analysis of Population Structure (BAPS). A) Structure plots of 205 isolates revealing 6 different major populations (Clusters 1–6). Phylogenetic species were assigned to each of the six deduced populations as follows: Cluster 1 representing the phylogenetic species NAm 1, Cluster 2 representing the phylogenetic species RJ, Cluster 3 containing LAm B, Cluster 4 constituted by phylogenetic species NAm 2, Cluster 5 constituted by LAm A1, LAm A2, BR1-4, and the paraphyletic low supported clades Eurasia, Unknown 1 and Unknown 2 and Cluster 6 containing Netherlands, Panama, Africa, Australia and BAC1. B) Bayesian population tree based on substructures of the 6 initial clusters deduced by BAPS. Gene flow is represented by mixture isolates that are annotated with brackets along the tree.

Based on phylogenetic and population structure analyses, three main species are found in the United States: NAm 1, NAm cat-associated and NAm 2 [40] **(Fig 1)**. Those species are found to be monophyletic and supported by bootstrap and posterior probability values. In addition, those species have unique haplotype structures as revealed by Median-Joining vector analysis for each individual locus **(S1 Fig)**. Interestingly, the NAm 1 and cat-associated species shared a single common ancestor, indicating that speciation could be driven by host-specific interactions. According to population structure using BAPS, both NAm 1 and NAm 2 phylogenetic species are distinct from Clusters 1 and 4, respectively **(Fig 2**). In addition, no admixture was found between these clusters in agreement with high *F*_*ST*_ values suggesting no gene flow between populations **(S2 Table)**. The isolate 2134 from Texas and the isolate 2761 from Alabama grouped with the LAm A2 and unknown populations, respectively. This suggests that either those patients were infected in Central-South America, or those species are found in North America. No geographic differentiation between populations was observed, and both NAm 1 and NAm 2 species co-occur in the endemic areas of United States (**S2 Fig**). According to Tajima’s and Fu’s test of selective neutrality (Tajima’s D = -1.79268; p<0.05, and Fu’s Fs = -6.50886; p<0.02), NAm 2 populations may have expanded after a recent speciation (**S3 Table**). The haplotype diversity between NAm 1 and NAm 2 also indicates different population dynamics. We observe a complex haplotype network of the *arf* locus within NAm 2 while the NAm 1 isolates are nested in a single haplotype (**S1 Fig**). While NAm2 underwent a recent population expansion, NAm 1 may be subject to disruptive selection driven by different hosts (cat and human). Sexual reproduction has been demonstrated with North American strains of *Histoplasma* [7]. In addition, recombination and genetic exchange has been shown to occur [38]. Sexual reproduction allows fungal populations to adapt to adverse or novel conditions, which results in the emergence of novel and potentially more virulent genotypes that are able to infect to new hosts [69].

### Latin America *Histoplasma capsulatum* populations

*Histoplasma capsulatum* isolates from Latin America contributed to the highest genetic diversity compared with other regions of the globe. In fact, South and Central America contributes to the majority of cases of histoplasmosis in the world [4, 18]. Although histoplasmosis is not a reportable disease, the elevated incidence of this mycosis among the HIV/AIDS population as well the reports of several outbreaks in immunocompetent patients suggests a high prevalence of this disease in Latin America [4, 18, 70, 71].

At least two populations are reported within Latin America: LAm A and LAm B [40]. Several reports have described distinct genetic clusters containing taxa from environmental and clinical samples from Brazil, various bat-associated samples from Mexico and clinical samples from Honduras [41–43]. However the fine-scale evolutionary relationships within and between the LAm A and LAm B have not been addressed. Phylogenetic and evolutionary analyses reveal complex genetic structure within the Latin American populations **(Fig 1)**. The LAm B phylogenetic species comprises a single haplotype complex for *ole1*, *tub1* and *H-anti* loci (**S1 Fig**). According to the Bayesian inference of the genetic structure in *Histoplasma* the LAm B clade comprises Cluster 3 using the admixture model **(Fig 2A**). However, phylogenetic analysis suggests genetic differentiation within this proposed phylogenetic species (**Fig 1**). The isolates from Argentina/Colombia (LAm B1) and Brazil (LAm B2 –Rio Grande do Sul and Espírito Santo states) are placed in two different monophyletic clades strongly supported by bootstrap and posterior probability values **(Fig 1)**. Moreover, a refined population structure of Cluster 3 suggests the same split. According to **Fig 2B**, LAm B1 and LAm B2 are represented by a single population cluster. Admixtures between LAm B clades and within Cluster 5 were also observed (**Fig 2B, S4 Table**). In addition to those observations, LAm B populations exhibit excess of high-frequency variants, indicating balancing selection (Tajima’s *D* = -1.51512; p<0.05) suggesting population expansion (LAm B1 and LAm B2) after recent speciation (**Fig 1, S3 Table)**. It is worth mentioning that the majority of LAm B1 isolates were isolated from HIV-infected patients (**Fig 1**).

The former LAm A clade exhibits greater genetic diversity and several phylogenetic species were revealed after inclusion of other Latin American isolates. The newly analyzed dataset revealed a large number of polytomic branches due to homoplastic characters, which suggests a rapid diversification within the former LAm A clade. We propose splitting the LAm A clade into at least 3 phylogenetic species (RJ, LAm A1 and LAm A2). Clades with low support such as Eurasia and Unknown2 were placed within the former LAm A clade (**Fig 1**). Two phylogenetic species represented by Central America and Colombia isolates were proposed: LAm A1 and LAm A2 (**Fig 1**). The phylogenetic species LAm A1 and LAm A2 are composed of isolates obtained in Mexico, Guatemala, Honduras, Panama and Colombia (**S1 Table**). Haplotype networks also support both phylogenetic species, especially for the *tub* locus where both are separated in haplotypes clusters (**S1 Fig**). Within LAm A1 the Colombian strains clustered in a cryptic monophyletic branch (LAm A1) along with a single Honduran strain indicating intraspecific genetic isolation (**Fig 1**). In addition, a cryptic branch (H141 clade) harboring the isolates H141 (Indonesia) and H188 (Panama) was also inferred. According to Tajima’s and Fu’s test of selective neutrality, LAm A1 (Tajima’s *D* = -2.18568; p<0.05 and Fu’s *F*s = -13.08523; p<0.02) and LAm A2 (Tajima’s *D* = -1.74401; p<0.05 and Fu’s *F*s = -2.13715; p<0.02) populations exhibit excess of high-frequency variants, from a large haplotype (Tajima’s *D* = -1.79268; p<0.05 and Fu’s *F*s = -6.50886; p<0.02) suggesting population expansion after a recent speciation from the basal former LAm A clade (**S3 Table)**. Interestingly, some monophyletic clusters were found among the Latin American phylogenetic species, which supports population expansion **(Fig 1)**.

Within the LAm A clade we detected a single monophyletic lineage of both environmental and clinical isolates collected in Rio de Janeiro and São Paulo state of Brazil, indicating geographic isolation (**Fig 1**). This cryptic species was designated RJ and is supported by both bootstrap and posterior probability values **(Fig 1)**. In addition, the RJ clade is represented by a single haplotype complex in the *H-anti* and *tub1* loci supporting the proposition of a cryptic species within the *H*. *capsulatum sensu lato* (**S1 Fig**). Moreover, the RJ isolates composed a unique population as revealed by Bayesian analysis and grouped in Cluster 2 (**Fig 2B**). Interestingly, both soil and clinical isolates cluster together, corroborating soil as the source of infection **(Fig 1)**. A number of supported monophyletic branches within RJ phylogenetic species are in agreement with population diversity under neutrality, since both Tajima’s *D* and Fu’s *F*s test indicated negative values, however not statistically significant, which indicates sequences are evolving randomly (-1.3065; p = 0.076 and -6.51844; p = 0.031 respectively; **S3 Table**).

Other monophyletic lineages were found within Brazilian isolates. There are four different monophyletic lineages (BR1-4) harboring isolates from Brazil that are supported by both bootstrap and posterior probability values found along the tree and are also supported by single haplotypes (**Fig 1**, **S1 Fig**). The BR1 clade is composed by two isolates (SP49 and H149) from São Paulo state in Brazil. The BR2 clade is composed by three isolates from Rio de Janeiro (84476, 84502 and 84564); one from São Paulo state (H151) and one from Ceará state in Brazil (JIEF) represented by single haplotypes in the *H-anti* and *tub1* loci. The BR3 clade is formed by an isolate from Rio de Janeiro (H196), São Paulo (H154) and Espírito Santo (ES62) and is also supported by a single haplotype within the *tub1* locus. Finally, the BR4 clade harbors three isolates: two from Pernambuco state (RE5646 and RE9463) and another one from Brazil but without identification (H146) and supported by a single haplotype within the *H-anti* locus **(Fig 1)**. Because of a small sample size, the neutrality tests did not indicate a significant effect (**S3 Table**). According to population structure analysis, isolates from the BR1-4 clades are admixtures between Cluster 5 and Cluster 2, suggesting that these groups are freely recombining (**Fig 2, S4 Table**). However, population structure analysis suggests admixtures between Cluster 5 and Cluster 2, suggesting gene flow within the former LAm A clade (**S4 Table**). In order to avoid misleading taxonomic classification we do not rank the BR1-4 clades as phylogenetic species due to potential recent hybridization within the well-supported Latin American phylogenetic species (**Fig 1, S1 Fig**). The Panama clade was also identified to be strongly monophyletic, supported and represented by an unique haplotype in the current analysis (**Fig 1, S1 Fig)** and no structural genetic differences within this clade were found even with additional Latin American strains [40]. Moreover the isolate EH315 from the BAC1 clade appears to be result of a hybridization event between Cluster 6 and Cluster 4 indicating that introgression may occur within this genus, allowing the emergence of new genotypes (**Fig 2, S4 Table**).

### Non-American *Histoplasma capsulatum* populations

On the African continent, histoplasmosis was first described in 1952 by Dubois and colleagues [72] and is also known as histoplasmosis *duboisii*, or African histoplasmosis caused by *Histoplasma duboisii*. The disease is prevalent in the tropical belt of Africa, more specifically in the western and central areas of the sub-Saharan, as well as Madagascar. About 300 histoplasmosis cases were reported up to 2007, mostly due the increase of the HIV-infected population, as well as antiretroviral therapy [35]. Imported cases of African histoplasmosis have been diagnosed in Europe [73]. Due to inefficiency and inaccuracy of diagnosis of deep mycosis in these areas, those numbers are likely to be an underestimate.

Histoplasmosis is reported frequently in horses throughout the African continent, but associated with *H*. *capsulatum* var. *farciminosum* [28, 29]. However, an analysis of equine cases revealed that the disease could be caused by other varieties of *Histoplasma* [40]. In addition, the former classification of *H*. *capsulatum* var. *duboisii* based on occurrence in Africa was determined to be obsolete, due to the fact that the *H*. *capsulatum* var. *duboisii* and *H*. *capsulatum* var. *capsulatum* phenotypic variants co-occur within Africa [40].

Previous phylogenetic analysis of African strains (*H*. *capsulatum* var. *duboisii* and *H*. *capsulatum* var. *capsulatum*) with the addition of three new genotyped isolates revealed a different genetic structure than previously demonstrated [40, 74]. Three groups (AF1-3) within Africa species were detected, which contained highly supported branches and single haplotypes (**Fig 1, S1 Fig**). Structure analysis places the Africa species within the Cluster 6 (**Fig 2A**). However, a more detailed analysis of Cluster 6 revealed that African isolates are represented by a unique genotype (phylogeny and haplotypes) but no interspecies differentiation was observed in the BAPS analysis (**Fig 2B**). Neutrality tests suggest that the African population of *Histoplasma* is under a balancing selection or has undergone a recent bottleneck or effective population size decrease (*D*>0 and *F*_*s*_>0), however they are not statically significant (**S3 Table)**. Despite analyzing few sequences, a potential population bottleneck was observed in the African clade. We suggest that the African continent could harbor more *Histoplasma* species and disease burden than reported.

The Eurasian clade contains isolates from North Africa (Egypt), Europe (England, German and Poland) and Asia (Thailand, China and India). A monophyletic clade was observed with the same isolate compositions previously reported, however with low bootstrap support [40] (**Fig 1**). Also, the Eurasian clade is represented by a single group within Cluster 5 (**Fig 2**). Of note is that almost all equine *Histoplasma* isolates cluster within the Eurasian clade, which may suggest susceptibility of horses to a particular genotype of *Histoplasma* **(Fig 1)**. The Netherlands and Australia phylogenetic species remain unaltered as no additional isolates were added. Both species are monophyletic and are represented by single haplotypes (**Fig 1, S1 Fig**). However, an intrapopulation analysis revealed that these two species compose a single cluster, cluster 6 (**Fig 2B**). Histoplasmosis should be further explored in Europe, Africa and Asia, especially with the increase of HIV cases. Serological surveys and PCR-based methods revealed the prevalence of the fungus in Asia with new potential regions of *Histoplasma* exposure [75, 76]. Also new cases and emergent genotypes have been recently identified in non-HIV patients from China [77]. Recently, disseminated histoplasmosis was diagnosed in a non-HIV patient from Sweden, which is considered a non-endemic region [78]. Our main conclusion from this limited sampling is that *H*. *capsulatum* is more widespread in non-endemic regions than previously thought [34, 37, 79], and thus more effort to understand both the ecology and distribution in these regions is warranted.

### Bat/bird flight’s potential role in *Histoplasma* speciation and global distribution

Beyond the Americas, *Histoplasma* is endemic to Africa, Asia, Europe and Oceania, however at a lower incidence based on reported cases [34, 35, 37, 80]. Among the Onygenalean mycoses, histoplasmosis caused by *H*. *capsulatum sensu* lato is rather unique. This species is found on 5 continents, and exhibits higher genetic variation when compared to *Coccidioides*, *Blastomyces* and *Paracoccidioides* [81]. *Histoplasma* has a worldwide distribution while *Paracoccidioides*, *Coccidioides*, and *Blastomyces* have limited geographic distribution [81, 82]. Similar to *Emmonsia* species that also cause systemic infections outside the Americas [83], *Histoplasma* is found on all continents, and therefore it is reasonable to hypothesize a mechanism of long-range dispersal.

One of the important questions concerning the evolution of *Histoplasma* species remains unanswered: Why does *Histoplasma* have a worldwide distribution compared to its close ajellomycetacean relatives? Some ecological features may reveal dispersion mechanisms of this pathogen. First, *Histoplasma* is found and grows in nitrogen/phosphate (bird and bat guano) enriched soils and environments, which selects against other microorganisms [2, 3]. Second, *Histoplasma* can naturally infect avian and chiropteran species, and disseminated disease is reported in those organisms [19, 84]. Third, some avian and chiropteran species are long-range migratory reservoirs, aiding in the dispersal of this microorganism [85].

*Histoplasma* is recurrently isolated from bats in endemic areas of histoplasmosis [42, 86]. The fungus was isolated from internal organs as well as in bat guano-enriched soils [42, 43]. Other closely related human fungal pathogens such as *Coccidioides posadasii* [87], *Blastomyces dermatitidis* [88], and *Paracoccidioides brasiliensis* [89] have been isolated only sporadically from bats. Bats are widely distributed across many continents and ecosystems, being one of the largest groups of mammals, second in number of species after the order Rodentia and first in number of individuals [90]. Bats live in caves, abandoned or occupied buildings, mines, plant crowns, bark, or rocks, which in association with humidity and guano enriched microenvironment favors the development of *Histoplasma* [86]. Some significant bat-associated clades were reported in our phylogenetic analysis, which reinforces that bats may drive speciation and aid in dispersal. Within the LAm A1 and LAm A2, two supported clades containing bat-derived *Histoplasma* strains were identified (**Fig 1**). Because of its guano-growth ability in sheltered habitats, *Histoplasma* likely may have a focal distribution, with habitats that can be widely separated from each other.

**Fig 3:**
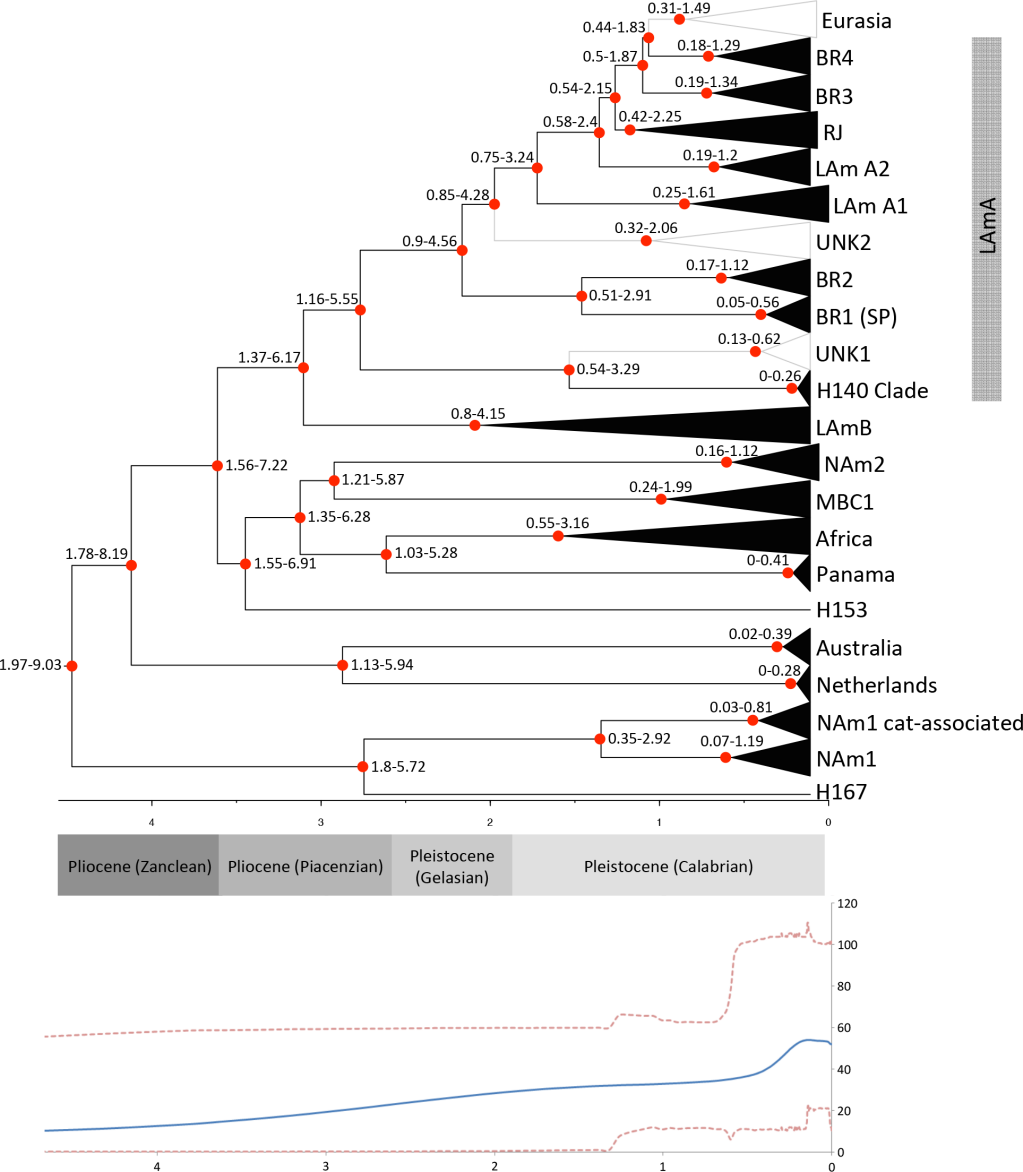
Extended Bayesian Skyline Plot (EBSP) of *Histoplasma capsulatum sensu lato*. EBSPs represents population size changes over time and divergence dating was inferred using BEAST v1.8.2 based on conservative intervals of nucleotide substitutions rates and dates (0.00043–0.00656 subst/site/lineage/My; 0.0–15 Ma) that encompass values obtained by Kasuga et al. [40]. Y-axes are effective population size divided by generation time. X-axes are in millions of years. Confidence intervals of each dated phylogenetic species were added to the nodes.

The LAm A1 bat-associated clade is composed of the strains EH375 and EH376 while the LAm A2 bat-associated is harbors the isolates EH372, EH373, EH377 and EH378. Both clades contain isolates recovered from *Artibeus hirsutus* (i.e., hairy fruit-eating) from Morelos-Mexico, indicating that bats could play an important role in the *Histoplasma* speciation and dispersion [42]. This is in agreement with population expansion revealed by neutrality tests performed within LAm A1 and LAm A2 populations. In addition, we designate a bat-associated species-specific clade (BAC1) harboring *Histoplasma* genotypes (EH384I and EH384P isolates) associated with the long-migratory bat species *Tadarida brasiliensis* as well as with *Mormoops megalophylla* (EH315 isolate) (**Fig 1**). This cryptic species harbors the isolates EH315, EH384I and EH384P as previously suggested by Taylor et al. [41, 42] and no human clinical isolates were placed within this clade so far. Recently, the addition of 6 new *Histoplasma* strains isolated from *T*. *brasiliensis* reinforced the proposition of a new phylogenetic species (Vite-Garín submitted). Bats originated in the early Eocene (52–50 Mya) coincident with the global temperature rise [91] and as well with the origin of ancestral *Histoplasma* [92], however the diversification of the *Histoplasma*-associated bats species such as the *Artibeus* genus occurred more recently. The radiation of *Artibeus* genus is estimated to have initiated around 13.2 Mya (19.9–8.8 Mya) in the Miocene, splitting into *Artibeus* and *Dermadura* clades followed by diversification [93]. This is in agreement with the subsequent radiation of the *Histoplama capsulatum* complex **(Fig 3**) around 1.97–9.03 Mya. Bats represent an interesting model to understand the dispersal of *Histoplasma* and its ability to infect humans; however, the mechanisms are still unknown. Interestingly, several studies indicate that bat mitochondrial physiology has evolved to tolerate oxidative stress incurred during high metabolic flights, possibly allowing tolerance to intracellular parasitism [94]. These results strongly suggest that *Histoplasma* could co-evolve with its most frequently associated host.

### Geological history of *Histoplasma*

The demographic history of *Histoplasma* was assessed by Extended Bayesian Skyline Plot (EBSP) analyses using relaxed molecular clock models for Bayesian inferences. Convergence of the Beast runs, ESSs, and “*burn-ins*” were >200 for all parameters. According to EBSP analyses and relaxed molecular clock models for Bayesian inferences, the radiation of *H*. *capsulatum sensu stricto* took place between 1.97 to 9.03 Mya during the Neocene period, more specifically from the late Miocene through the Pleistocene epochs (**Fig 3**). The fact that the highest genetic diversity was found in Latin America, as well as ancestral branches/lineages such as H167 and H153, strongly suggests that the center of dispersion of *Histoplasma* was in fact South/Central America (**Figs 1 and 2**, [40]). In addition, the EBSP tree clearly shows that diversification of *Histoplasma* occurred about 0.5 Mya during the Pleistocene epoch. According to population tree, two main events of radiation took place during *Histoplasma* evolution. The first radiation event is clearly observed in the “old world” and probably originated from the basal H153 genotype as well as the NAm 1, NAm 2, Africa, Netherlands, Australia and Panama/BAC1 species (**Fig 2**). It is important to note that this speciation took place between 1.97–9.03 Mya, and these new populations remained stable as revealed by long branch lengths and low diversity (**Figs 2 and 3**), except for the North American clades which originated more recently and harbor a higher genetic diversity (**S3 Table**). There are two main possibilities for the long-range *Histoplasma* colonization: intercontinental macro/microconidia migration via air dispersal or by long-range migratory animals. *Histoplasma* can survive passage through the digestive tract of bats [94–96] and some avian species [3, 19, 84]. The second radiation of *Histoplasma* took place more recently (1.37–6.17 Mya). The short branches associated with the new emerging lineages, high genetic diversity, population expansion (deduced by neutrality tests), and high rates of recombination within and between groups are in agreement with the emergence of new Latin American genotypes **(Figs 1–3**). This recent radiation took place in Latin America and appears to be a constant process that according to the EBSP analysis occurred about 0.5 Mya **(Fig 3)**. Those genotypes could be associated with more virulent/pathogenic variants that could reflect in different disease phenotypes. Recently, studies of host-pathogen interactions using a small set of *Histoplasma* isolates (NAm1, NAm2, Panama and LAm A species) revealed significant differences in fungal burden, disease kinetics, disease symptoms, and cytokine response [97, 98].

Summarizing, based on the concordance between GCPSR and population structures methods, six new phylogenetic species are proposed: RJ, LAm B1, LAm B2, LAm A1, LAm A2, BAC1 **(Figs 1 and 2)**. The clades Eurasia, BR1-4, Unknown 1 and Unknown 2 were not concordant among the three addressed methods and gene flow was found between these isolates and the RJ, LAm B1, LAm B2, LAm A1 and LAm A2 phylogenetic species. Many processes, such as incomplete lineage sorting, horizontal gene transfer, gene/genome duplication, recombination and hybridization can all create incongruities within closely related organisms [99, 100]. In order to resolve those incongruences, a whole genome sequencing approach to define and evaluate a broader number of SNPs across the *Histoplasma* population is suggested. Strong evidence of host-derived speciation and radiation in *Histoplasma* species was found. The radiation of both *Histoplasma* and its more frequently associated bat host, *A*. *hirsutus*, took place in similar geographical areas **(Fig 3**). Moreover, well-supported clades of bat-associated isolates were detected, reinforcing the hypothesis that bats may play an important role in diversification and dispersion of *Histoplasma capsulatum*.

Assessing pathogen genotypes will provide the foundation to understand the variable clinical manifestations of this important fungal disease. For example, are there specific *Histoplasma* genotypes responsible for outbreaks or severe clinical cases in immunocompetent humans? Are there isolates with differential sporulation, expression of virulence factors, or tolerance to extreme nitrogen/phosphate enriched soils? Does speciation affect the sequence or expression of any reported virulence genes in *Histoplasma* such as *hsp60/82*, *yps3*, *ryp1/2*, *cbp1* or *ags1* [101, 102]? Further studies to explore the genetic diversity of *Histoplasma* and correlate genotype with medically important phenotypes via population genomics and genome wide association studies [103] will help provide answers to the above questions.

## Supporting Information

S1 Fig Median-joining networks showing haplotype distribution of *Histoplasma capsulatum sensu*. Diagrams were inferred through haplotypes from 4 different loci as follows: *arf*, *ole1*, *tub1* and *H*-*anti*. Circles are proportional to haplotype frequency and numbers of haplotype (h) and Haplotype Diversity index are shown. Black dots represent a single mutation and red dots represent median-joining vectors.
(PDF)

S2 Fig Population frequency and distribution of *Histoplasma capsulatum sensu stricto* overlaid with the epidemiological map of histoplasmosis in United States. Epidemiological map was obtained at the Centers for Disease Control and Prevention (CDC) web site: www.cdc.gov/fungal/diseases/histoplasmosis/causes.html.
(PDF)

S1 Table List of *Histoplasma* strains used in the present work. (XLSX)

S2 Table Pairedwise ΦST estimates for the main *Histoplasma* populations. (XLS)

S3 Table Neutrality tests within *Histoplasma* populations. (XLSX)

S4 Table Mixture results of BAPS population clusterization of *Histoplasma*. (XLS)
