# Supplementary figures and images for: Worldwide Phylogenetic Distributions and Population Dynamics of the Genus Histoplasma
Source: PLoS Negl Trop Dis. 2016 Jun 1;10(6):e0004732. doi: 10.1371/journal.pntd.0004732 (PMC4889077; doi:10.1371/journal.pntd.0004732)

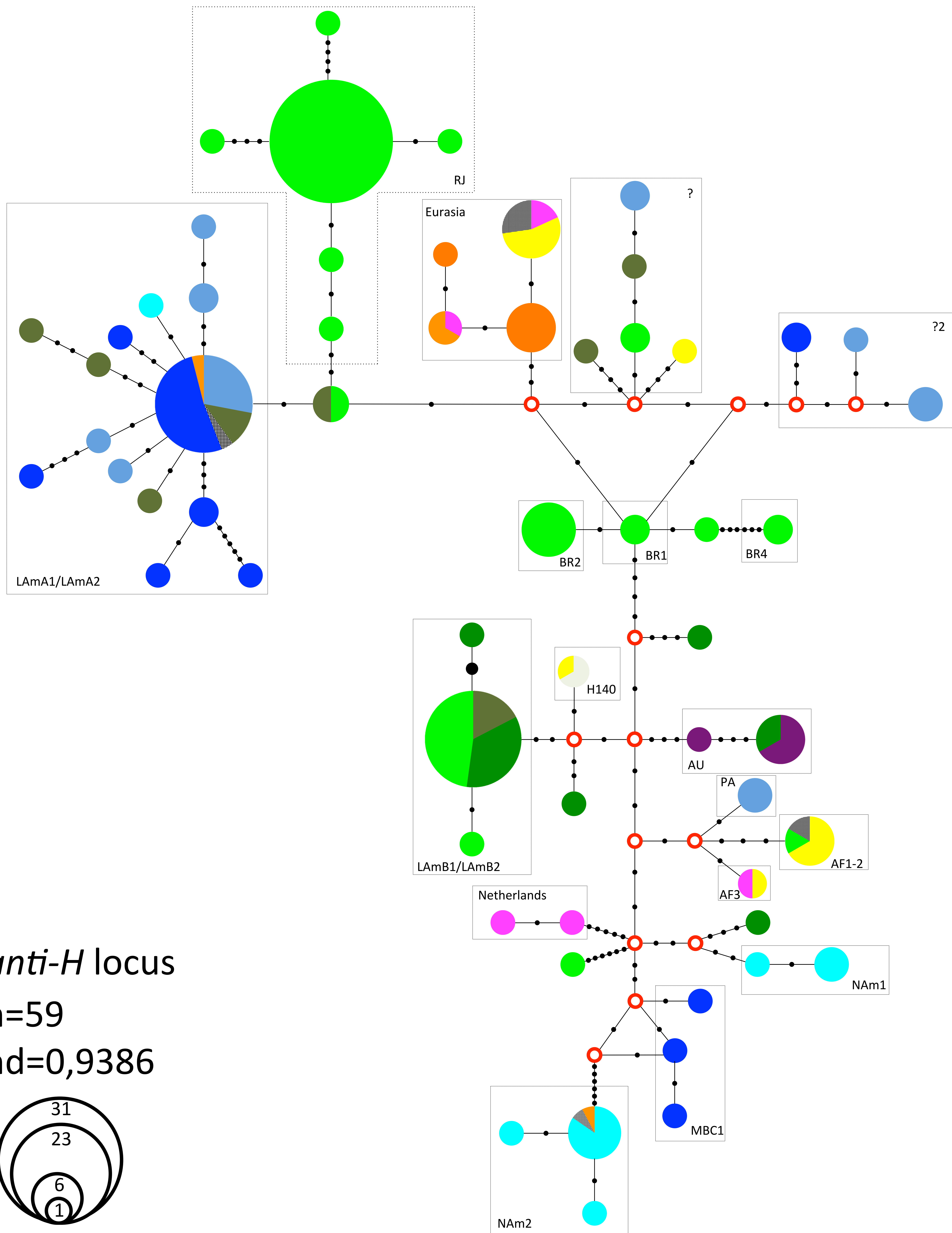

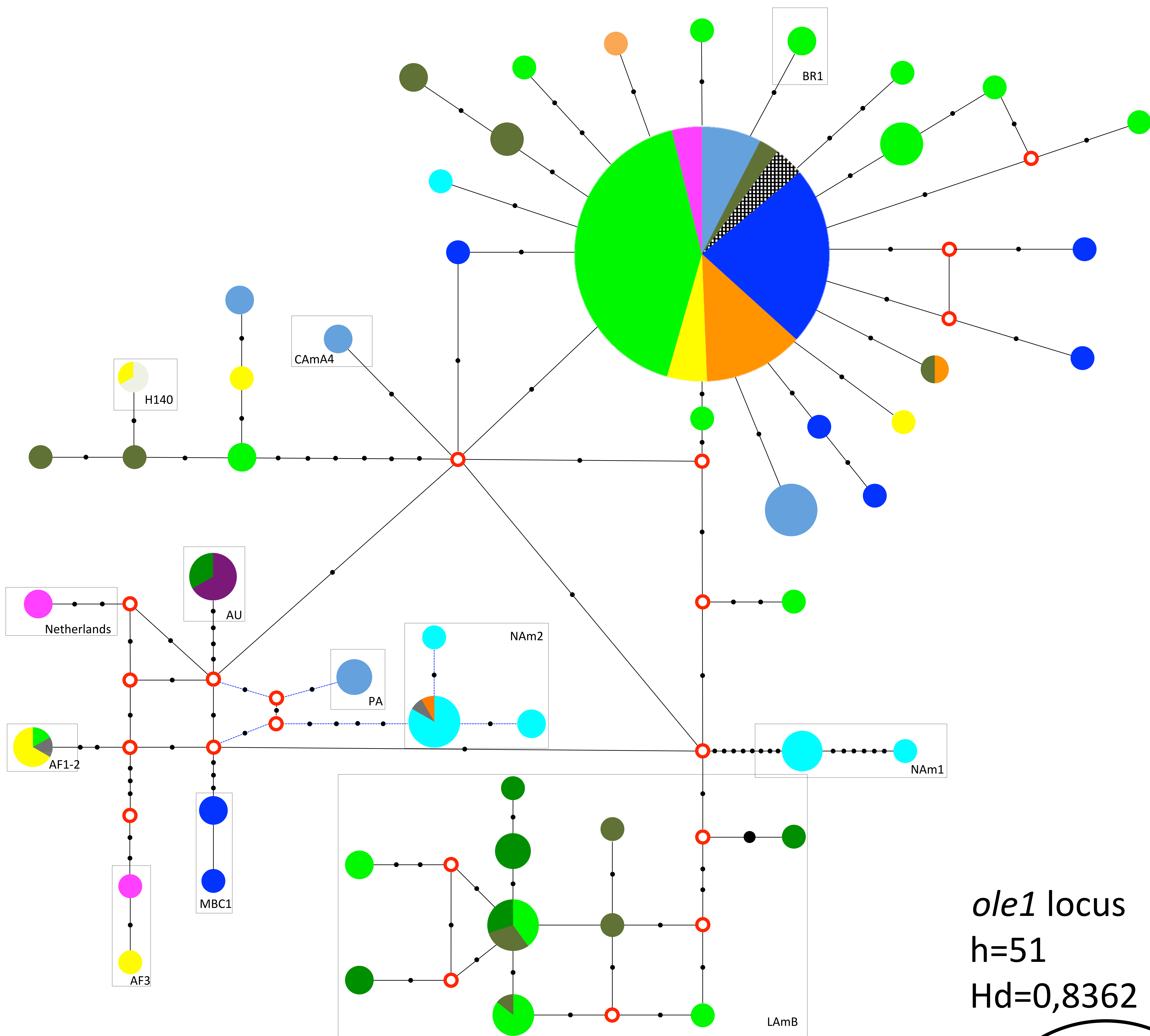

*ole1* locus  
 $h=51$   
 $Hd=0,8362$

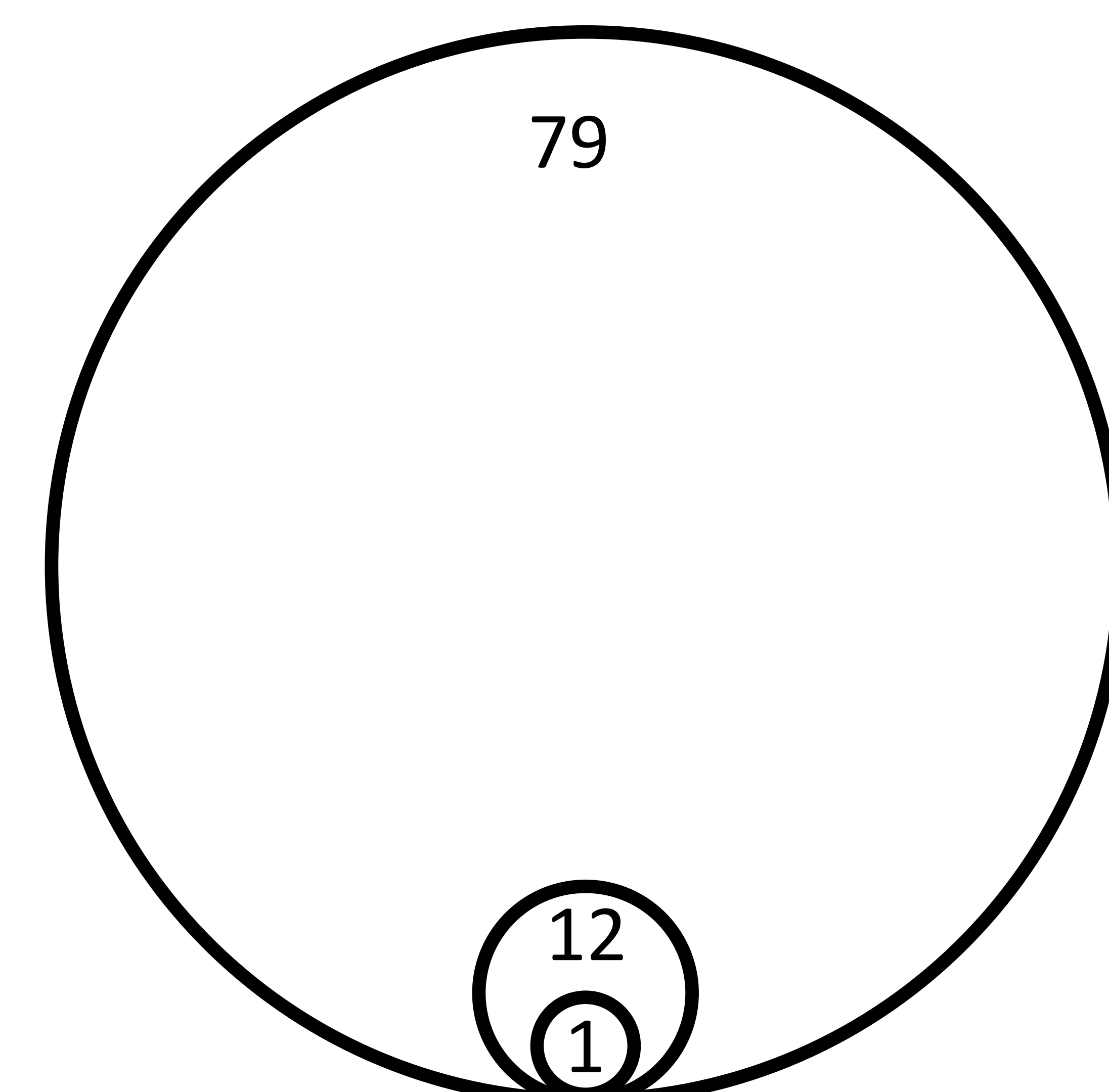

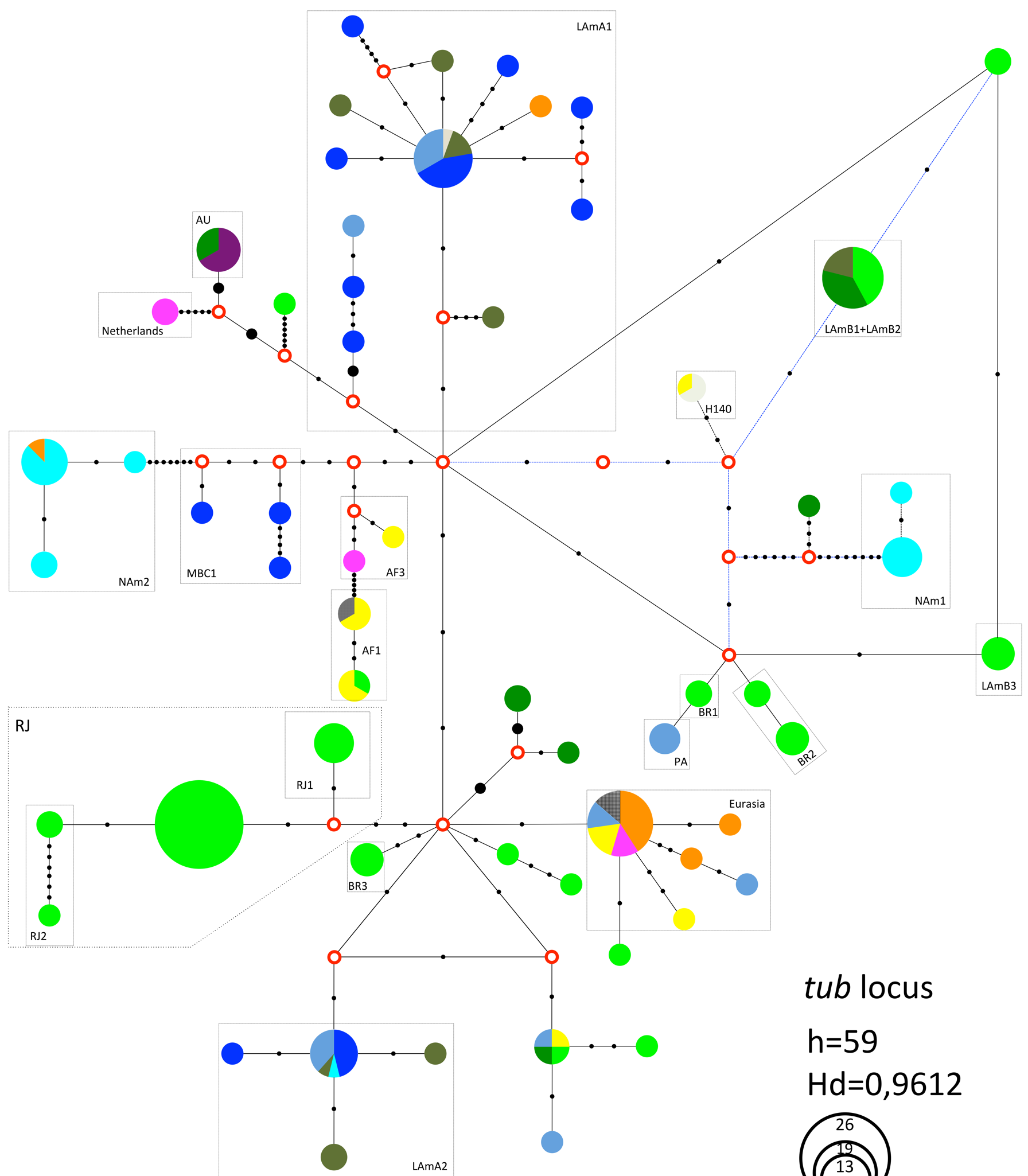

*tub* locus

h=59

Hd=0,9612

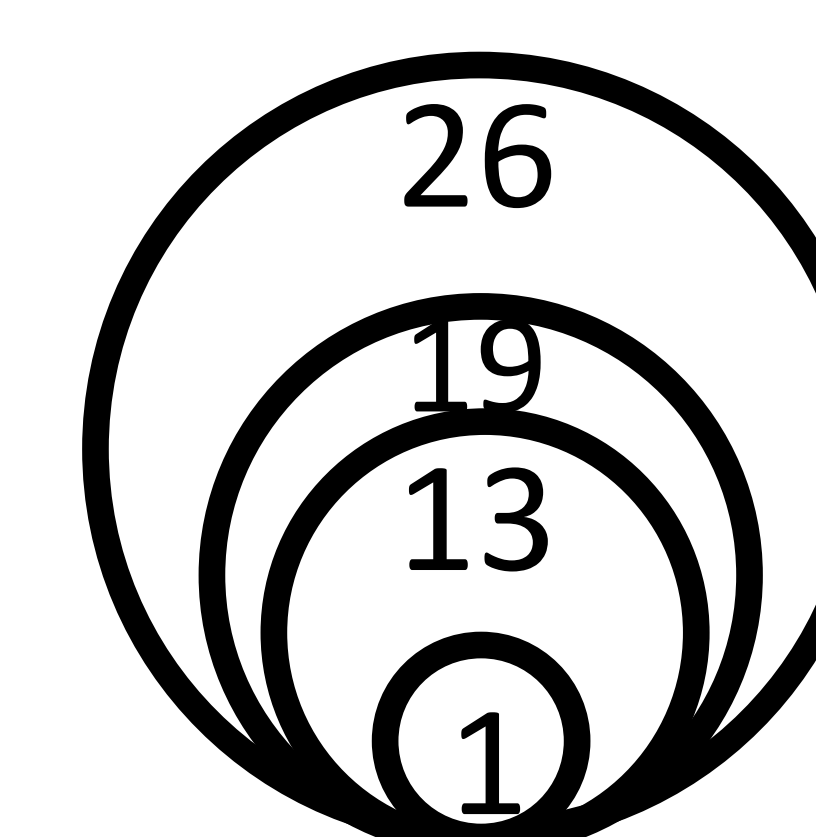

Supplement: S1 Fig — Diagrams were inferred through haplotypes from 4 different loci as follows: arf, ole1, tub1 and H-anti. Circles are proportional to haplotype frequency and numbers of haplotype (h) and Haplotype Diversity index are shown. Black dots represent a single mutation and red dots represent median-joining vectors. (PDF) [file pntd.0004732.s001.pdf]

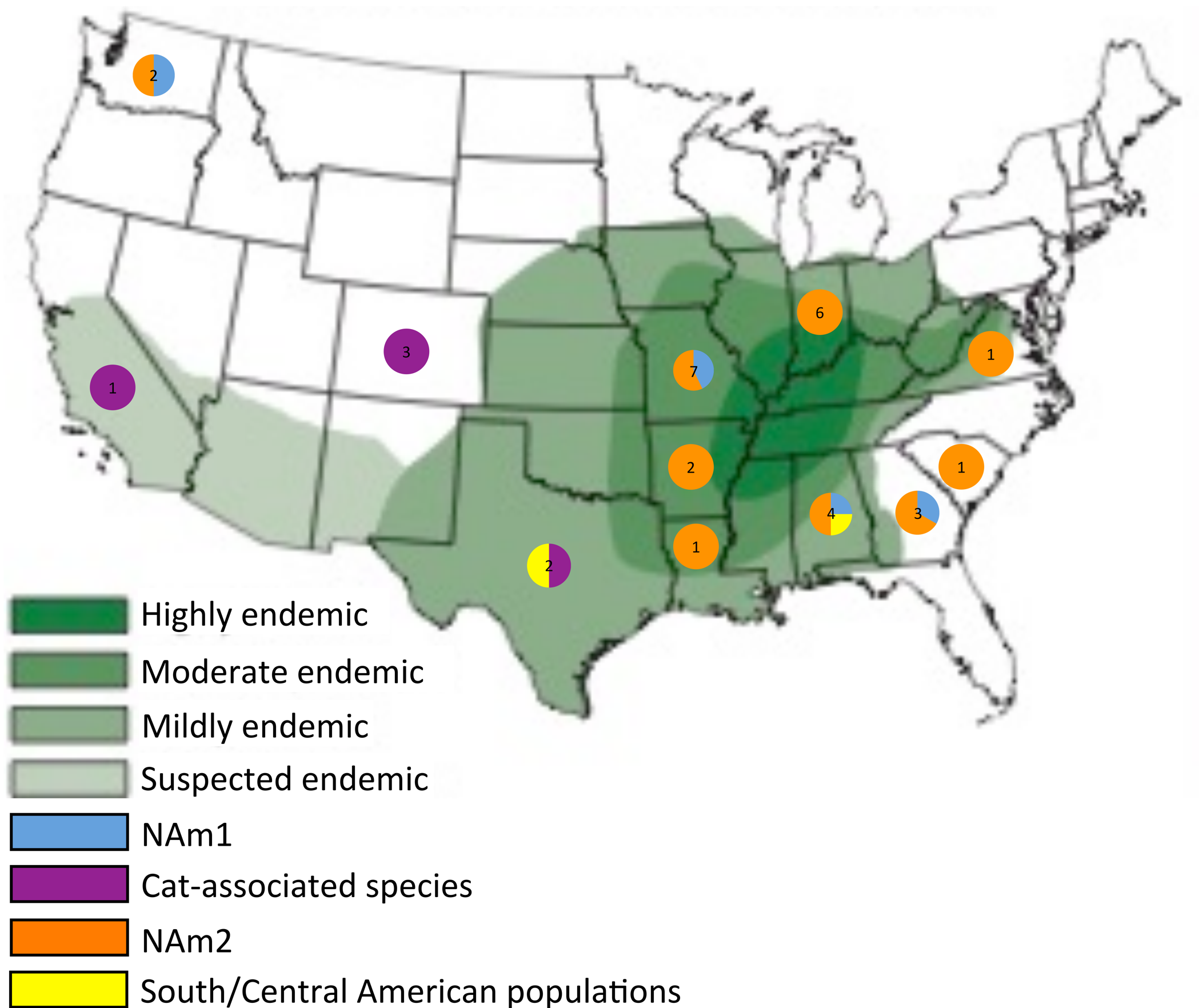

Supplement: S2 Fig — Epidemiological map was obtained at the Centers for Disease Control and Prevention (CDC) web site: www.cdc.gov/fungal/diseases/histoplasmosis/causes.html. (PDF) [file pntd.0004732.s002.pdf]
